# The OWL Reasoner Evaluation (ORE) 2015 Competition Report

**DOI:** 10.1007/s10817-017-9406-8

**Published:** 2017-02-21

**Authors:** Bijan Parsia, Nicolas Matentzoglu, Rafael S. Gonçalves, Birte Glimm, Andreas Steigmiller

**Affiliations:** 1 Information Management Group, University of Manchester, Manchester, UK; 2 Stanford Center for Biomedical Informatics Research, Stanford University, Stanford, CA USA; 3 Institute of Artificial Intelligence, University of Ulm, Ulm, Germany

**Keywords:** OWL, Ontologies, Reasoning

## Abstract

The OWL Reasoner Evaluation competition is an annual competition (with an associated workshop) that pits OWL 2 compliant reasoners against each other on various standard reasoning tasks over naturally occurring problems. The 2015 competition was the third of its sort and had 14 reasoners competing in six tracks comprising three tasks (consistency, classification, and realisation) over two profiles (OWL 2 DL and EL). In this paper, we discuss the design, execution and results of the 2015 competition with particular attention to lessons learned for benchmarking, comparative experiments, and future competitions.

## Introduction

The Web Ontology Language (OWL) is in its second iteration (OWL 2) [13, 29] and has seen significant adoption especially in Health Care (see, e.g., [20, 38]) and Life Sciences (see, e.g., [5, 12, 48]). OWL 2 DL can be seen as a variant of the description logic (DL) ${\mathcal {SROIQ}}$ [2, 18]. The three *profiles* introduced in OWL 2 (called OWL EL, OWL QL, and OWL RL) [26] correspond to logical fragments of ${{\mathcal {SROIQ}}}$ and were designed to allow for a more simple or efficient implementation. Finally, OWL 2 Full is a syntactic extension of OWL 2 DL that does not correspond to a description logic.

Description logics generally are designed to be *computationally practical* so that, even if they do not have tractable worst-case complexity for key services, they nevertheless admit implementations that seem to work well in practice [10]. Unlike the early days of description logics or even of the direct precursors of OWL (DAML+OIL [6]), the reasoner landscape [22, 37] for OWL is rich, diverse, and highly compliant with a common, detailed specification. Thus, we have a large number of high performance, production-quality reasoners with similar core capacities (with respect to language features and standard inference tasks).

Research on optimising OWL reasoning continues apace, though empirical work still lags behind both theoretical and engineering work in breadth, depth, and sophistication. There is, in general, a lack of shared understanding of test cases, test scenarios, infrastructure, and experiment design. A common strategy in research communities to help address these issues is to hold competitions, that is, experiments designed and hosted by third parties on an independent (often constrained, but sometimes expanded) infrastructure. Such competitions, in contrast to published benchmarks, do not always provide in depth empirical characterisations of the competing tools. Instead, they serve two key functions: (1) they provide a clear, motivating event that helps drive tool development (e.g., for correctness or performance) and (2) *components* of the competition are useful for subsequent research. Finally, competitions can be great fun and help foster a strong community. They can be especially useful for newcomers by providing a simple way to gain some prima facie validation of their tools without the burden of designing and executing complex experiments themselves.

Toward these ends, we have been running a competition for OWL reasoners (with an associated workshop [3, 4, 7, 15]): the OWL Reasoner Evaluation (ORE) competition [33]. ORE has been running, in substantively its current form, for 3 years. In this paper we describe the 2015 competition (held in conjunction with the 28th International Description Logic Workshop (DL 2015)^1^ in June 2015. The competition comprises two different components: the live competition, the heart of ORE, pits a number of competing reasoners against each other on a carefully crafted corpus of OWL ontologies, featuring a timeout of 3 min and a single run; and the offline competition, which features particularly reasoning intensive ontologies submitted by the ontology engineering community and runs with a 6 h timeout per ontology and reasoner. An overview of all resources (reasoners, ontology corpus, competition result data and analysis scripts, competition framework) is also available online.^2^

The contribution of this paper consists of a discussion of the general competition design and execution as well as a summary of the results of the 2015 competition with particular attention to lessons learned for benchmarking, comparative experiments, and future competitions. While many log files and statistics of the competition are publicly available, the aggregated results and their analysis, as presented in this paper, provide in-depth insights that are otherwise quite time-consuming to obtain. The description of the competition framework allows developers to easily rerun the competition with new or updated reasoners to get a sense of their relative progress. The discussion of the competition design fosters a shared understanding of test cases, test scenarios, infrastructure, and experiment design within the DL community. The ORE 2015 corpus, which we describe in this paper, is a significant and distinct corpus for reasoner experimentation whether used with the ORE framework or in a custom test harness. The ORE toolkit and corpora may further serve as a nucleus for an infrastructure for common experimentation. Some of the lessons learned might inspire competition organisers in other fields or communities who want to establish a competition for their research area.

The remainder of this paper is organised as follows: we next introduce some preliminaries regarding OWL. Section 3 introduces the overall competition design, the compilation of the used ontology corpus, a description of the ontologies contributed by users, the framework to run the competition and the used technical environment. Section 4 describes the participating systems. Sections 5 and 6 introduce the setup and outcome of the live and the offline competition, respectively. Finally, we conclude in Sect. 7 with a summary of the competition results and some challenges that should be addressed in future competitions.

## Preliminaries

Before we describe the competition set-up, we first give brief introduction to OWL as relevant for the remainder of the paper. For a full definition of OWL 2, please refer to the OWL 2 Structural Specification and Direct Semantics [28, 29].

A domain of interest can be modelled in OWL 2 by means of *individuals* (which denote objects from the domain of discourse), *literals* (which denote data values, such as strings or integers), *classes* (which denote sets of individuals), *datatypes* (which denote sets of data values), *object properties* (which relate pairs of individuals), and *data properties* (which relate individuals with concrete values). Individuals, classes, datatypes, and object properties can be used to form *class expressions*, *data ranges*, and *object property expressions*, respectively; these are complex descriptions of sets of individuals, sets of literals, and relationships between individuals. Finally, class expressions, data ranges, object property expressions, data properties, individuals, and literals can be used to form *axioms*—statements that describe the domain being modelled. Axioms describing individuals are commonly called *assertions*. An OWL 2 *ontology* *O* is a finite set of axioms.

The semantics of axioms in an OWL ontology *O* is given by means of two-sorted interpretations over the *object domain* and the *data domain*, where the latter contains well-known data values such as integers and strings. An *interpretation* *I* maps individuals to elements of the object domain, literals to elements of the data domain, classes to subsets of the object domain, datatypes to subsets of the data domain, object properties to sets of pairs of object domain elements, and data properties to sets of pairs whose first component is from the object domain and whose second component is from the data domain. An individual *i* is an *instance* of a class *C* in an interpretation *I* if the image of *C* contains the image of *i*. An interpretation *I* is a *model* of an ontology *O* if *I* satisfies all conditions listed in [28]. For example, if *O* contains an axiom stating that *C* is a subclass of *D*, then the conditions from [28] require each instance of *C* in *I* to also be an instance of *D* in *I*. If the axioms of *O* cannot be satisfied in any interpretation (i.e., if *O* has no model), then *O* is *inconsistent*; otherwise, *O* is *consistent*. If the interpretation of a class *C* is contained in the interpretation of a class *D* in all models of *O*, then *C* is a *subclass* of *D* (or, equivalently, *D* *subsumes* *C*) in *O*. If the interpretation of an individual *i* is contained in the interpretation of a class *C* in all models of *O*, then *i* is an *instance* of *C* in *O*.

Conventionally, the set of axioms is divided into two parts, the TBox and the ABox. The TBox comprises concept definitions and inclusions and corresponds to the “schema” part of the ontology. The ABox is a collection of ground assertions which corresponds to the “data” part of the ontology. Each part has characteristic reasoning tasks, e.g., classification for the TBox and instantiation for the ABox.

## Competition Design

The ORE competition is inspired by and modelled on the CADE ATP System Competition (CASC) [34, 44] which has been running for 25 years and has been heavily influential in the automated theorem proving community^3^ (especially for first-order logic).

We observe that central to such competitions is participation, thus various incentives to participate are critical especially in the early years of the competition as it is trying to get established. Hence the importance of “fun” elements, incentives (e.g., prizes, bragging rights), as well as a reasonable chance of winning at least *something*.

The key common elements between ORE and CASC are:

1. A number of distinct tracks/divisions/disciplines characterised by problem type (e.g., “effectively propositional” or “OWL 2 EL ontology”).
2. The test problems are derived from a large, neutral, updated yearly set of problems (e.g., for CASC, the TPTP library [43]).
3. Reasoners compete (primarily) on how many problems they are able to solve within a given timeout.

As description logics have a varied set of core inference services supported by essentially all reasoners, ORE also has track distinctions based on task (e.g., classification or realisation). Other CASC inspired elements:

1. The reasoner ranking is derived solely from a *live competition run* during the Description Logic workshop, i.e., the offline performance evaluation across user submitted ontologies does not feed into the ranking.
2. There was a secondary competition among DL attendees to predict the results for various reasoners.
3. Competitors and organisers were given T-shirts designed specifically for the event, where the design goes beyond the typical printing of event names and logos.

### Tracks

ORE 2015 had six tracks based on three central reasoning services (consistency, classification, and realisation) and two OWL profiles (OWL DL and EL). These services are not ubiquitously supported, with realisation not handled by some reasoners. We use the following definitions for these services (though any consequence equivalent definition would do):

- *Consistency* checking is the task of determining whether an ontology $\mathcal {O}$ is *consistent* or not.
- *Classification* is the task of computing all entailed class subsumptions between named classes in the ontology.
- Ontology *realisation* refers to computing all entailed class assertions for named classes and individual names occurring in the ontology, i.e., the computation of all instances for all named classes in the ontology. This tasks is also known as *materialisation*.

Consistency is, in some sense, the most fundamental service. Classification is, almost certainly, the most common and important reasoning service for ontologies to date. Realisation gets us at least a minimal form of instance reasoning.

We aim to extend the competition by other OWL profiles when we have enough participants that are specifically tuned for that profile. In prior years we also had an RL track, but the number of RL-specific reasoners is very low. We hope to introduce a conjunctive query track in future years and discuss some of the challenges in Sect. 7. All reasoners purporting to handle the entirety of OWL 2 DL are entered in all tracks. Thus, we have specialised EL reasoners competing against fully-fledged OWL DL reasoners.

For each track, we award prizes to the top three participants for a total of 18 possible winners. Awards are only given for the winners of the live competition. The offline competition is aimed at informing the ontology and reasoner developers of potential issues as well as engaging the ontology development community. For reasoner developers, the offline competition typically feature harder and logically expressive ontologies which have proven troublesome for users. For ontology developers, in addition to bringing their ontologies in view of reasoner developers, they have their ontologies tested on a wider range of reasoners in a robust setting.

### Live Competition Corpus

The full live competition corpus contains 1920 ontologies. Each competition comes with its own random stratified sample of ontologies from this base corpus for the live competition—that is not all 1920 ontologies are actually used in a live competition. The competition corpus is sampled from three source corpora: a January 2015 snapshot of Bioportal [30] containing 330 biomedical ontologies, the Oxford Ontology Library^4^ with 793 ontologies that were collected for the purpose of ontology-related tool evaluation, and MOWLCorp [21], a corpus based on a 2014 snapshot of a Web crawl containing around 21,000 unique ontologies.

The ontologies in the corpus were pre-processed using the OWL API (v3.5.1) [14]. As a first step, the ontologies of all three source corpora were collected and serialised into OWL/XML with their imports closure merged into a single ontology. The merging is, from a competition perspective, necessary to mitigate the bottleneck of loading potentially large imports repeatedly over the network, and because the hosts of frequently imported ontologies sometimes impose restrictions on the number of simultaneous accesses.^5^ After the collection, the entire pool of 21,465 ontologies was divided into three groups: (1) Ontologies with less than 50 axioms (12,927 ontologies), (2) OWL 2 DL ontologies (4199), and (3) OWL 2 Full ontologies (4339). The first group was removed from the pool.

As reasoner developers could tune their reasoners towards the ontologies in the three publicly available source corpora, we included a number of approximations into our pool. The entire set of OWL 2 Full ontologies were approximated into OWL 2 DL, i.e., we used a (slightly modified) version of the OWL API profile checker to drop DL profile-violating axioms so that the remainder is in OWL 2 DL [23]. Because of some imperfections in the “DLification” process, this process had to be performed twice. For example, in the first round, the DL expressivity checker may have noted a missing declaration *and* an illegal punning. Fixing this would result in dropping the axiom(s) causing the illegal punning *as well as* injecting the declaration—which could result again in an illegal punning.

**Fig. 1:**
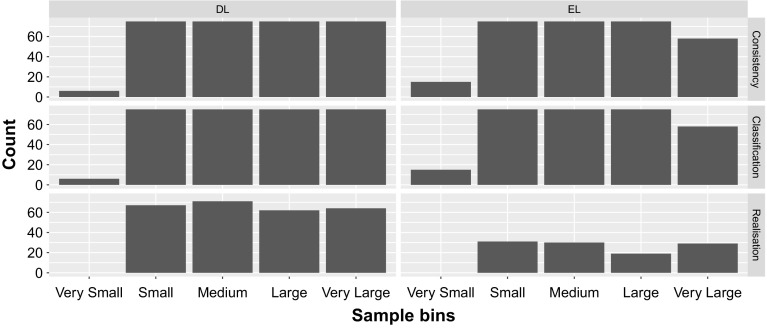
The number of ontologies sampled for each size bin

The OWL 2 DL group was then approximated using the OWL 2 EL/QL approximation method employed by TrOWL  [35]. This resulted in a 8644 successful approximations. As the only syntax that is uniformly supported by all reasoners participating in the competition, we serialised the entire pool (including the original OWL 2 DL ontologies, the approximated ontologies, and the “DLified” OWL 2 Full ontologies) into Functional Syntax, and gathered all relevant ontology metrics again. As some ontologies are included in more than one of the source corpora, we excluded at this point (as a last pre-processing step) all duplicates^6^ from the entire pool of ontologies and removed ontologies with TBoxes containing less than 50 axioms. The random stratified sampling for the competition then was done as follows: All ontologies were binned by size into the following groups: Very small (50–99 axioms), small (100–999 axioms), medium (1000–9999 axioms), large (10,000–100,000 axioms) and very large (more than 100,000 axioms). From each group, we attempted to sample 60 original ontologies, and 15 approximated (i.e., the “ELified” and “DLified”) ontologies for each competition. For the OWL 2 EL related tracks, the ontologies had to fall under the OWL 2 EL profile, for the OWL 2 DL competitions, the ontologies had to fall under OWL 2 DL but *not* under any of the three OWL 2 profiles, and for the two realisation challenges we only considered those ontologies that had at least 100 ABox axioms. This process resulted in the following six live competition corpora: 306 for OWL DL Consistency and Classification, 264 for OWL DL Realisation, 298 for OWL EL Consistency and Classification, and 109 for OWL EL Realisation. Figure 1 shows the results of the sampling, i.e. the number of ontologies for each bin.

The full competition corpus (1920 unique OWL 2 DL ontologies), and the execution order of the competition, can be obtained from Zenodo [24].

### User Submitted Ontologies

The offline competition corpus consists of ontologies submitted by users. For ORE 2015, we had four user submissions consisting of a total of seven ontologies. The user submissions underwent the same pre-processing procedures as the corpus (Sect. 3.2). This occasionally had large consequences on the ontologies, most importantly with respect to rules (they were stripped out) and any axiom beyond OWL 2 DL (for example, axioms redefining built-in vocabulary or violating the global constraints on role hierarchies, see [23]). Therefore, the results of the offline competition cannot serve as the final answer to the question of which reasoner is the best for the respective ontology developers, but will hopefully give direction. The user-submitted corpus has two parts: one containing the submissions for ORE 2015, and one for the user submissions of 2014.^7^ We will only provide a detailed break-down of the results for the 2015 corpus, and present the aggregated results for the 2014 corpus. The following ontologies were submitted to ORE 2015:

- *Cell Ontology* (CO):^8^ CO is designed as a controlled vocabulary for cell types. It is not organism specific, covering various cell types from mammals to prokaryotes.^9^
- *Drug-Drug Interactions Ontology* (DINTO):^10^ DINTO is a pharmacological ontology that systematically organises drug-drug interaction (DDI) related knowledge that contains pharmacological substances, proteins and relationships among them. DDIs are represented at class level. The submission contained five versions of DINTO. Unfortunately, ORE reasoners are not required to deal with SWRL rules; therefore, only the basic (and pre-processed) version of DINTO was admitted to the competition.^11^
- *Drosophila Phenotype Ontology* (DPO):$^{8}$ DPO was designed as a queryable classification of phenotypes from the FlyBase knowledge base [31].^12^
- *Gene Ontology Plus* (GO-PLUS):$^{8}$ GO-PLUS is the fully axiomatised public release of the Gene Ontology. It includes axioms referencing classes from multiple external ontologies.^13^
- *Virtual Fly Brain Ontologies* (VFB):$^{8}$ Three of the VFB ontologies were submitted: VFB-KB, combing the Drosophila anatomy ontology (DAO) with a knowledge base of anatomical individuals, VFB-EPNT, an experimental extension of DAO including spatial disjointness axioms for the adult brain, combined with a knowledge base of expression patterns including explicit negation, and VFB-NCT, an experimental extension of DAO including spatial disjointness axioms for the adult brain as well as closure axioms on the synapsing patterns of neurons (i.e., this is in the DL profile of OWL).^14^

All ontologies submitted to ORE 2015 are proper OWL DL ontologies, i.e., they do not fall into any of the OWL 2 profiles. Metrics regarding the number of axioms and the used description logic (expressivity) for the ontologies can be found in Table 1. Seeing as only 3 of the submitted ontologies contain ABox axioms (the VFB variants), these were the only ontologies tested in the realisation track.

**Table 1:** Breakdown of user-submitted ontologies in the ORE 2015 corpus

Ontology	TBox	ABox	Ontology	TBox	ABox
CO	7527	0	VFB-EPN	33,612	63,295
DINTO	123,930	0	VFB-KB	20,187	147,996
DPO	917	0	VFB-NCT	33,612	63,295
GO+	150,955	0			

The submissions from ORE 2014 include the following:

- City Benchmark (CB)^15^ [8].
- Data Mining Ontologies (DMOP)^16^ [17].
- USDA and DPC^17^ [49].
- Some variants of GALEN and FMA.
- The Genomic Clinical Decision Support Ontology (G-CDS)^18^ [36].
- The Family History Knowledge Base (FHKB)^19^ [42].

A complete list of the ontologies submitted in 2014 with metrics regarding the number of axioms in the ontologies is presented in Table 2. CB-BERN, CB-CORK, and CB-VIENNA fall into the OWL 2 QL profile, FTC belongs to the OWL 2 EL profile, and all other ontologies are proper OWL DL ontologies, i.e., they do not fall into any of the OWL 2 profiles.

**Table 2:** Breakdown of user-submitted ontologies in the ORE 2014 corpus

Ontology	TBox	ABox	Ontology	TBox	ABox
CB-BERN	428	209,932	MSC-D	9532	0
CB-CORK	428	20,393	MSC	9532	318
DCHARS	1925	1728	FHKB-V3	425	3307
DGO	233	47,603	RMO-A	1925	15,759
DMKB	1925	1606	FHKB-V1	355	3296
DMOP	1986	765	DPC-OLY	122	35,866
DPC-1	122	54,898	PD	1930	973
DPC-2	122	79,955	FHKB-V2	419	3304
FMA-CPFNS	123,024	86	USDA10	174	3602
FTC	140,799	0	USDA15	176	5948
GALEN-FU	37,411	0	USDA20	176	8600
GALEN-H	10,628	0	USDA25	177	9785
G-CDS	4322	0	USDA5	174	1226
G-CDS-D	4322	140	CB-VIENNA	428	584,266
HP	123	17,027			

### The Competition Framework

The competition framework used in ORE 2015 is a slightly modified version of the one used for ORE 2014, which is open sourced under the LGPL license and available on Github.^20^

The framework supports both serial and parallel execution of a competition. With serial execution or serial mode, we refer to running the competition on a single computer, where the reasoners are run one after the other on all problems. Parallel execution or parallel mode means that the competition is configured to run on a cluster of computers, where one master machine dispatches evaluation tasks (i.e., evaluating a reasoning task for a specific reasoner on a given ontology) to client machines, collects the results and serves them up to a live display. Parallel (distributed) mode is used for the live competition, but serial mode is sufficient for testing or offline experiments. The framework also logs sufficient information to allow “replaying” the competition, and includes scripts for a complete replay as well as directly showing the final results.

The framework is realised with Java and, therefore, it should be runnable on all Java supported platforms. Reasoners are required to parse and serialise OWL’s functional-style syntax [29]. This syntax is designed to allow for easy processing and was supported by all participating reasoners. In order to run a reasoner within the framework, reasoner developers have to provide a script (a shell script and, optionally, a Windows batch script) that can be used to start the reasoner with parameters to indicate the input ontology and the task that is to be performed. Reasoners also report processing times, results, and processing errors via the invocation script. Apart from reported processing errors (e.g., a reasoner stops processing an ontology due to encountered unsupported datatypes), the framework also records crashes, e.g., due to the memory limit, as errors in log files. Finally, the framework produces log files to record timeouts and wrong results. Reasoners have to report results of a reasoning task in a specific output format that allows for an easy comparison (using hash codes) of the reported result with an expected one. Furthermore, the script is used to enforce the given time and memory limits.

Since many reasoners support the Java-based OWL API, there is a standard script for OWL API based reasoners and a Java wrapper class that implements the functionality for producing the desired result outputs and for error handling. This makes it easy to prepare reasoners with OWL API support for the competition and we explicitly encourage OWL API support as it supports access to the reasoners by a plethora of tools. OWL API support is, however, not required to participate in the competition. The OWL API is a very rich and rather heavyweight framework that is not tightly integrated with most reasoners. For example, systems using the OWL API generally consume more memory because they maintain the OWL API level representation of the ontology in addition to the internal representation of the reasoner. Thus, avoiding the OWL API can help competition performance. Furthermore, for reasoners not written in Java OWL API support can be difficult or time-consuming to implement. Using a script instead of Java code to start the reasoners allows for easy integration of reasoners not implemented in Java or without OWL API support.

The framework uses configurable timeouts for each reasoning task assessed in the competition. For reasoners that exceed the time limit set for a competition, the ulimit command is used to enforce termination. The reasoners report the time needed to solve a problem themselves in wall clock time.

*Methodological Aspects* It was decided to measure the times in wall clock time instead of CPU time, because CPU time would penalise parallel reasoners such as ELK. Recording CPU time in addition to the wall clock time is, however, a noteworthy extension of the current framework. The time measurement is performed by the reasoners and the current specification “recommends” excluding the time for “standard” parsing and loading as well as the time needed for result serialisation (i.e., writing the results to output files). The idea behind this is to not punish reasoners that offer very flexible parsing (and serialisation) support of all kinds of syntaxes, e.g., by using the rich but heavyweight OWL API for this task, over those that have a slim, specialised parser that just processes the easy-to-handle functional-style syntax. In addition, reasoners that employ specialised parsers such as ELK, ELepHant, and Konclude often perform some kind of reasoning (e.g., whether a consistency check can be omitted because the ontology does not use negation), indexing, and pre-processing already during parsing. This makes it difficult to clearly separate loading and reasoning time. Hence, ELK chooses to always include loading times in the reported time, while Konclude does this for consistency checking, where the amount of reasoning time is much less dominating than for the other tasks. As far as we know, all other reasoners do not include parsing/serialisation time for any reasoning task. With the exception of ELepHant and Racer, these systems are implemented in Java and simply use the OWL API for which the parsing/serialisation times are easily separable. Furthermore, the current framework utilises a network drive to enable the reasoners access to the relevant files (e.g., the ontology documents). Hence, read and write operations can be influenced by the workload of the network and should be excluded or separated in the evaluation results.

For Java-based reasoners the JVM overhead might be a disadvantage due to the “fire and forget” execution strategy employed by the competition framework. This would particularly affect “easy” problems that do not require significant computations and running time. By using a long running server based approach the JVM overhead for easy cases could be effectively amortised.

In the current competition set-up, the reasoner-reported times have, however, a limited influence. They are only used for ranking the reasoners that solved an equal number of problems.

Results are validated by comparison between competitors using a majority vote/random tie-breaking fallback strategy. This dispute resolution mechanism is clearly unsatisfactory. Recent work [19] has revealed examples in the 2015 corpus where the correct reasoner would be unfairly penalized for being in the minority. Especially problematic are two facts: (1) The votes of deliberately incomplete (with respect to their purported profile) reasoners such as TrOWL can outweigh votes of a complete reasoner in the voting procedure. (2) Reasoners might be able to vote several times. For example, HermiT participated in two versions (one using OWL API version 3 and one using version 4) and, furthermore, it is used in the coalition reasoner MORe. Hence, a bug in HermiT might result in three reasoners delivering the same wrong result, which could outweigh two other correct reasoners. A similar problem potentially arises for Jfact and FaCT++, as Jfact is an (almost) faithful Java port of FaCT++. Note, unlike CASC, reasoners are not required to produce proofs of their results as this is not a standard feature of description logic reasoners. Note that for many services (such as classification) proofs for all subsumptions would be needed. Furthermore, (tableau-based) reasoner construct finite representations of infinite models and it is yet unclear how such partial models can be represented in a form that allows for verifying them automatically. We are, however, experimenting with a more satisfactory justification-based technique for disagreement resolution [19] in future competitions.

### Competition Environments

*Live Competition* The competition was run in parallel mode on a cluster of 19 machines: one master machine that dispatched reasoners with problems to the 18 client machines, as well as collecting and serving up results to a live display. Each machine was equipped an Intel Xeon quad-core L5410 processor running at 2.33 GHz with 12 GB of RAM, for which 2 GB were reserved for the operating system (i.e., 10 GB could be used by the reasoners). The operating system was Ubuntu 14.04.02 LTS and the Java version was OpenJDK v1.7.0 64-bit. The reasoner execution was limited to 180 s for each ontology in each track, where only 150 s were allowed for reasoning and 30 s could additionally be used for parsing and writing results in order to reduce the penalisation of reasoners with slow parsers. Hence, if the time reported by the reasoner exceeded 150 s, then it was interpreted as a timeout. These time limits were chosen such that the live competition could be run within 1 day (parallel to the DL/ORE workshop program) on the given hardware with a reasonable number of ontologies (200–300).^21^

*Offline Competition* The offline competition for user-submitted ontologies was run on an Amazon EC2 cluster where twenty instances were used, one of which was the master machine, running the competition server, and the remaining nineteen were client machines. The Amazon EC2 instances used were of type “r3.large”, with the following specifications: dual-core Intel Xeon E5-2670 (v2) processor running at 2.5 GHz clock speed, and with 15 GB of RAM memory, out of which 2 GB were reserved for the operating system, and the remaining 13 GB were available for reasoners. The operating system was Ubuntu Server 14.04 LTS, and the Java version was OpenJDK v1.7.0 64-bit. The reasoner execution time was limited to 6 h and 10 min for each ontology, where 6 h were allowed for reasoning and the additional 10 min could be used for input–output operations, following the same rationale as the live competition described above. We know from previous experiments that classification on Amazon EC2 instances is reasonably stable (i.e., low average variance), so each task was run only once. Running the competition multiple times would have consumed considerable computational resources for only a marginal gain—a single run took around 75 machine-days, i.e. it took ten machines (run in parallel) more than a week to execute the competition.

## Competition Participants

There were 14 reasoners participating, with 11 purporting to cover OWL 2 DL, and 3 being OWL 2 EL specific (see Table 3). There is no specific penalty or test for being incomplete with respect to a profile and, indeed, one reasoner (TrOWL) is intentionally incomplete for performance reasons.

The number of participants has been fairly stable over the past 3 years, ranging from 11 to 14. There is a stable core of participants with some fluctuation on the margin. Some reasoners are not entered by their original developers (e.g., Pellet) and ORE currently has no policy against that. We anticipate in the future that more coalition reasoners will be made available, though currently only MORe, Chainsaw, and PAGOdA use component reasoners (ELK and HermiT are used by MORe, FaCT++ by Chainsaw, and RDFox [27] and HermiT by PAGOdA) that are mostly also competing. For example, MORe ’s coalition involves partitioning the ontology into an EL and DL part, dispatching each part to the respective tuned reasoner, and combining the results [1]. Coalition reasoners that do not transform the ontology in any relevant way will need special consideration if they were to participate.

**Table 3:** Participant list with OWL 2 DL reasoners in the top and OWL 2 EL reasoners in the bottom part

Reasoner	New 2015	Consistency	Classification	Realisation	Language	License
OWL DL						
Chainsaw [47]	–	$\checkmark$	$\checkmark$	$\checkmark$	Java	LGPL 2.0
FaCT++ [46]	–	$\checkmark$	$\checkmark$	$\checkmark$	C++	LGPL 2.0
HermiT $^\mathrm{a}$ [9]	–	$\checkmark$	$\checkmark$	$\checkmark$	Java	LGPL 3.0
Jfact [32]	–	$\checkmark$	$\checkmark$	$\checkmark$	Java	LGPL 2.0
Konclude [41]	–	$\checkmark$	$\checkmark$	$\checkmark$	C++	LGPL 2.1
MORe [1]	–	$\checkmark$	$\checkmark$	–	Java	LGPL 3.0
PAGOdA [50]	$\checkmark$	–	–	$\checkmark$	Java	Academic license
Pellet-OA4 [40]	$\checkmark$	$\checkmark$	$\checkmark$	$\checkmark$	Java	AGPL v3
Racer [11]	$\checkmark$	$\checkmark$	$\checkmark$	$\checkmark$	LISP	BSD 3-clause license
TrOWL [45]	–	$\checkmark$	$\checkmark$	$\checkmark$	Java	AGPL v3
OWL EL						
ELepHant [39]	–	$\checkmark$	$\checkmark$	$\checkmark$	C++	Apache Licence 2.0
ELK [16]	–	$\checkmark$	$\checkmark$	$\checkmark$	Java	Apache Licence 2.0
jcel [25]	–	$\checkmark$	$\checkmark$	–	Java	Apache Licence 2.0

$^\mathrm{a}$ HermiT was submitted with OWL API 3 and OWL API 4 bindings

In the following, we will introduce the participating reasoning systems. Much of the information presented here can be found online^22^ as well as in our recently conducted OWL reasoner survey [22]. The version information reflect the state of the system as it was submitted to ORE 2015. 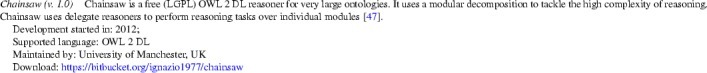 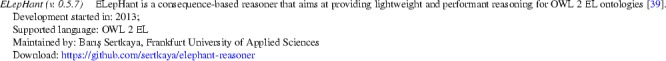 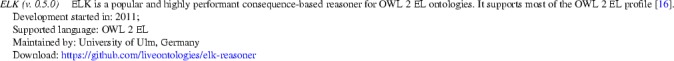 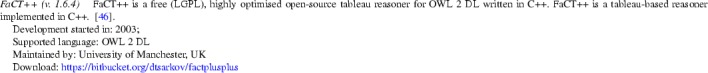 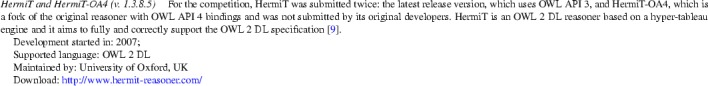 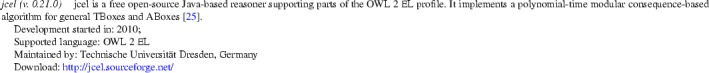 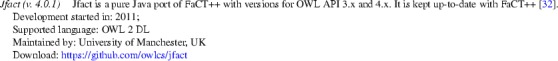 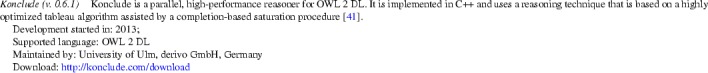 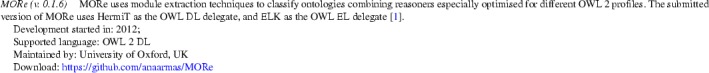 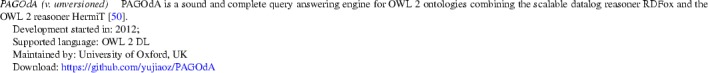 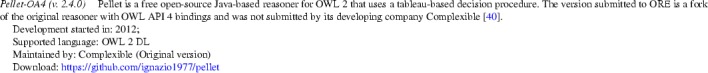 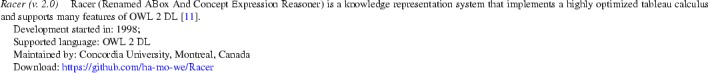 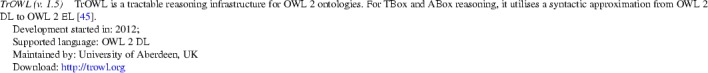


## Results: Live Competition

**Fig. 2:**
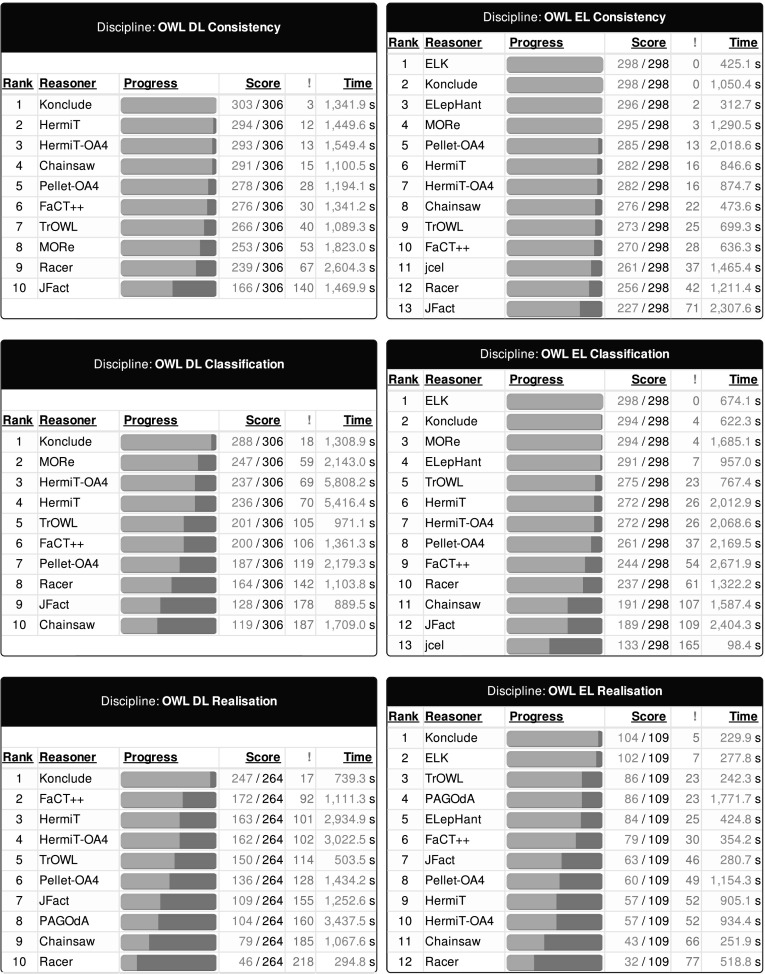
Results of the competition by track as displayed in the live competition display. *Score* indicates the number of problems solved out of the total problems for that track. The number of unsolved problems (whether by timeout, crash, or “wrong” results) are displayed in the *next column*. *Time* indicates the time actually taken to complete *solved* problems. *Time* is used to resolve ties for solved problems

Results, error reports, and more details on the competition framework are available at http://dl.kr.org/ore2015. A break-down of all tracks and the numbers of competing reasoners is shown in Table 4. Figure 2 shows the results of all participants in all tracks, as displayed during the live competition. During the competition, these charts were dynamically updated as problems were being solved and reported. Note that due to space constraints the error column (labelled with an exclamation mark) shows the sum of the number of errors, timeouts, and unexpected (wrong) results produced by the reasoner, i.e., the number of (processed) ontologies that are not considered as correctly solved.

**Table 4:** Breakdown of the competition by track

Task	Competitors	Problems
OWL DL		
Consistency	10	306
Classification	10	306
Realisation	10	264
OWL EL		
Consistency	13	298
Classification	13	298
Realisation	12	109

It is worth noting that for the OWL EL tasks there are several ties in the number of solved problems. In this case the reasoning time as reported by the reasoners is taken to rank the reasoners. For OWL EL Consistency there is a tie between ELK (first place) and Konclude (second place), where ELK was determined the winner due to its lower accumulated reasoning time (425.1 s for ELK vs 1050.4 s for Konclude). In this case both reasoners include parsing time into the measured time and, hence, the ranking seems fair. Another tie occurs between HermiT (sixth place) and HermiT-OA4 (seventh place) with 846.6 and 874.7 s, respectively. It is not surprising that both versions of HermiT perform similarly and since loading times are not taken into account for both versions the ranking seems fair also in this case. For OWL EL Classification there is a tie between Konclude (second place) and MORe (third place) with 622.3 and 1685.1 s, where both reasoners exclude loading time from the reported times. As for classification, there is again a tie for HermiT (sixth place) and HermiT-OA4 (seventh place). Finally, there is a tie for TrOWL (third place) and PAGOdA (fourth place) with 241.3 and 1771.7 s, respectively.

**Fig. 3:**
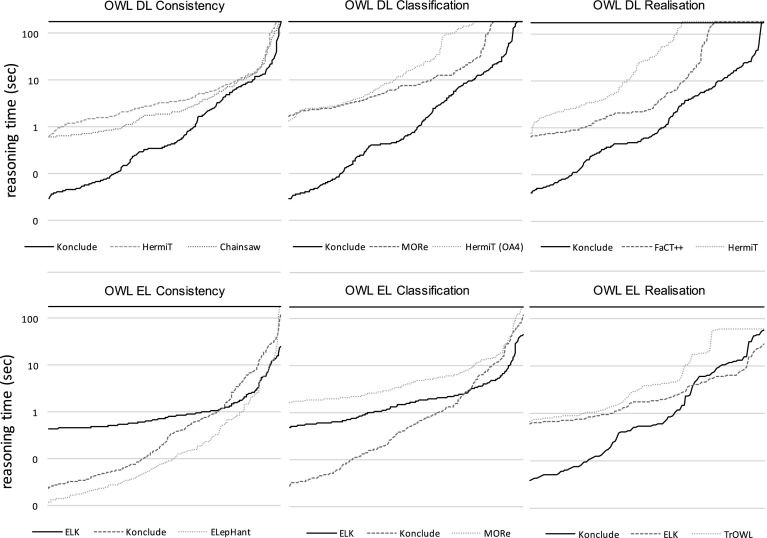
Reasoning time of the three winning reasoners in each category for the DL and EL profile (ordered separately by speed of the reasoner). The *top horizontal line* illustrates the timeout of 180 s

Out of the six tracks, four were won by the new hybrid reasoner Konclude  [41], and two (OWL EL Consistency and OWL EL Classification) were won by ELK  [16]. Figure 3 shows how well the winning reasoners did in terms of reasoning time. There are a couple of observations to be made here. First, Konclude, the winner of all three DL disciplines, is doing consistently better on the majority of the easier ontologies, but towards the harder end on the right, other reasoners catch up. This is particularly obvious for the EL classification competition. Up until a certain point, Konclude is doing much (sometimes up to an order of magnitude) better than ELK (the winner of the discipline), but towards the harder end, ELK overtakes Konclude. Some of this may be due to the JVM overhead for ELK and our “fire and forget” execution strategy. If we had a long running server based approach it might be that the JVM overhead for easy cases would be effectively amortised. Another interesting observation is the performance of ELepHant ’s [39] consistency check, which regularly outperforms both ELK and Konclude. We speculate that this is due to differences in whether parsing time is incorporated in the reported time (e.g., ELK does this for all tasks and Konclude does this for consistency checking).

A full break-down for all reasoners by competition is shown in Table 5.

**Fig. 4:**
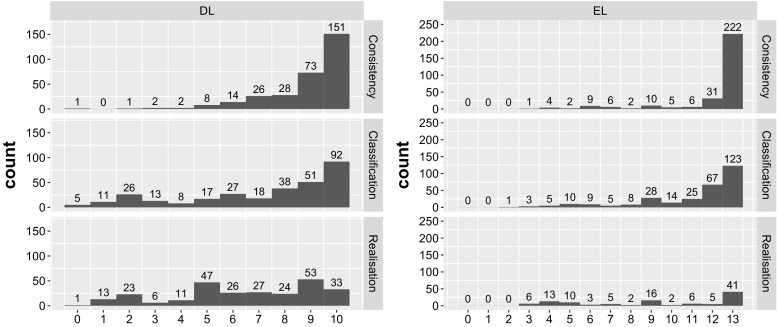
Number of reasoning problems (y-axis) by total number of reasoners solving them. For example, 5 DL classification tasks were not solved by any reasoner, and 123 EL classification tasks were solved by all reasoners

The competition is reasonably challenging: in only two tracks (EL consistency and EL classification) did any reasoner solve all the problems in competition conditions. Figure 4 shows a detailed breakdown of how many problems were solved by how many reasoners.

It is interesting to observe that the union of all reasoners successfully process all EL reasoning problems. As one might expect, realisation is still challenging for reasoners. But in all tracks, for the majority of reasoners, the ORE problems provide a good target for optimisation. The results of the competition suggest that these problems are (almost) all in principle solvable on a modest machine such as the ones used in our competition (see Sect. 3.5) in around 3 min.

The small number of (possibly) wrong results in the EL tracks further shows that reasoning with EL ontologies already achieved a good degree of stability and maturity. This also results in the fact that the majority voting is working quite well for the EL disciplines (to the best of our knowledge, for all EL Consistency and EL Classification problems, the correct results were determined). In contrast, there is much more disagreement on the DL tracks, which is due to several reasons. On the one hand, reasoning procedures for OWL 2 DL are much more involved and require many optimisations to work sufficiently well in practice. Hence, it can be difficult to ensure that implementations do not contain bugs. On the other hand, DL ontologies often contain datatypes in a way that affects reasoning, but several DL reasoners have only partial datatype support and thus may not derive all consequences. Furthermore, there are reasoners (e.g., TrOWL) that approximate more expressive language features and are, therefore, more likely to compute an incomplete result (for the more expressive DL ontologies). As a consequence, the majority voting can identify wrong results as correct and it is indeed likely that this happened in a few cases. This also seems to be indicated by the number of ties, which are 2 for the DL Consistency track, 13 for DL Classification, and 5 for DL Realisation (for the EL tracks, there were only 3 ties in the realisation discipline). Interestingly, most of the ties were between Konclude and TrOWL for “hard” DL ontologies that could not be solved by other systems.

**Table 5:** Full break-down of solved problems by reasoner and task over the 306 ontologies for DL consistency and classification, 264 for DL realisation, 298 for EL consistency and classification, and 109 for EL realisation

Reasoner	Solved	Timeout	Error	Wrong	Solved	Timeout	Error	Wrong
	DL consistency				EL consistency			
Chainsaw	291	3	11	1	276	19	3	0
ELepHant	–	–	–	–	296	2	0	0
ELK	–	–	–	–	298	0	0	0
FaCT++	276	16	13	1	270	22	6	0
HermiT	294	8	3	1	282	16	0	0
HermiT-OA4	293	8	4	1	282	16	0	0
jcel	–	–	–	–	261	35	2	0
Jfact	166	83	52	5	227	71	0	0
Konclude	303	1	0	2	298	0	0	0
MORe	253	43	2	8	295	3	0	0
Pellet-OA4	278	26	0	2	285	13	0	0
Racer	239	48	1	18	256	40	0	2
TrOWL	266	0	36	4	273	0	25	0
	DL classification				EL classification			
Chainsaw	119	171	16	0	191	94	13	0
ELepHant	–	–	–	–	291	6	0	1
ELK	–	–	–	–	298	0	0	0
FaCT++	200	87	17	2	244	51	3	0
HermiT	236	67	2	1	272	26	0	0
HermiT-OA4	237	66	2	1	272	26	0	0
jcel	–	–	–	–	133	158	6	1
Jfact	128	106	59	13	189	89	2	18
Konclude	288	7	1	10	294	0	0	4
MORe	247	41	2	16	294	2	0	2
Pellet-OA4	187	105	14	0	261	28	9	0
Racer	164	86	2	54	237	38	0	23
TrOWL	201	0	35	70	275	0	23	0
	DL realisation				EL realisation			
Chainsaw	79	166	16	3	43	64	2	0
ELepHant	–	–	–	–	84	1	0	24
ELK	–	–	–	–	102	0	0	7
FaCT++	172	58	25	9	79	27	3	0
HermiT	163	93	5	3	57	52	0	0
HermiT-OA4	162	93	6	3	57	52	0	0
Jfact	109	89	47	19	63	43	0	3
Konclude	247	2	1	14	104	0	0	5
PAGOdA	104	51	95	14	86	15	0	8
Pellet-OA4	136	54	24	50	60	32	2	15
Racer	46	75	3	140	32	31	0	46
TrOWL	150	0	43	71	86	0	22	1

## Results: Offline Competition

The live competition has quite strict time constraints as it must finish within a workshop day. Given the time limit of 150 s per ontology, it is not possible to include really hard ontologies in the competition. There are, however, ontologies used in real-world scenarios that are still challenging state-of-the-art reasoners. To address this, ORE allows users to submit their challenging ontologies to the offline competition, which has a much more generous time limit of 6 h, and a higher maximum memory (13 GB instead of 10 GB). The results of the offline competition are particularly valuable for reasoner developers as many optimisations are inspired by real-world problems. Furthermore, knowing which reasoners are able to handle some input, while others fail to, encourages discussions between developers regarding strategies and optimisation techniques.

The results of the 2015 offline competition are shown in Tables 6 and 7. The first thing to note is that at least one reasoner successfully processed each submitted ontology, for all three tasks. Konclude was the only reasoner that successfully terminated on every input. The CO ontology was particularly challenging for reasoners (see Table 6)—only 3 did not time-out during classification: FaCT++, Konclude and TrOWL. TrOWL classifies CO surprisingly fast, though it returns wrong results; FaCT++ and Konclude agreed on a different result to TrOWL. VFB-KB was another challenging ontology (see Table 7), where only Konclude correctly determined consistency without timing out or erring. Racer seemingly completed consistency checking on VFB-KB, but the result file did not contain the expected result; this is a recurring pattern for Racer and its *wrong* results in Tables 6 and 7.^23^ HermiT-OA4 and Jfact (seemingly) completed classification of VFB-KB, but erred or timed-out (respectively) during consistency checking over the same input. However, the results output by the 2 reasoners were incorrect; there were no subsumptions in the results files when there are, from manual inspection, inferences to be computed. Explaining such behaviour is beyond the scope of the competition. Note that the interfaces for classification and stand-alone consistency are different, so a bug affecting one may not necessarily affect the other. For Racer, checking consistency of VFB-KB (741.89 s) was only slightly faster (by 27 s) than computing classification—unfortunately, however, leading to the wrong result. It is interesting to note that HermiT-OA4 successfully completed classification of GO+ in less than half the time than HermiT. This may have happened due to insufficient or other memory problems, seeing as the more recent version of the OWL API boasts various memory improvements. This also occurs during consistency checking over the same ontology.

A striking result in Table 6 is that, while some reasoners were fast on the consistency task, such as Konclude that successfully terminated on every input or Chainsaw and TrOWL in all but one input, others did not even complete the consistency check within the 6 h timeout. Similar to the classification task, determining whether CO is consistent was challenging; half the reasoners timed-out, while the remainder terminated within 8 s at most. Even more challenging than CO, only one reasoner successfully completed consistency checking of VFB-KB; Konclude (3.81 s). Out of the remaining reasoners, four timed-out and the other five erred (where Racer seemingly completed the task, but reported an error and output empty results). On the other end of the spectrum, the only ontology for which every reasoner successfully terminated consistency checking was DPO, typically within 4 s (with the exception of MORe, which took 44.74 s).

Out of the three 2015 submissions that contained an ABox (see Table 7), one was notably demanding: VFB-KB, on which only two reasoners were able to successfully complete realisation within the timeout: Pellet-OA4 and Konclude, in under 9 s.

Unfortunately, the error information available was limited to that piped out to the specified hook (in the competition framework) for reasoner error output. From what we could determine the issues with the 2015 user-submitted corpus were mostly due to ontology parsing and internal reasoner errors while processing input. Apart from the fact that not all reasoners make use of the OWL API, parsing errors can occur at various points in the parsing process. The OWL API does not check several aspects, e.g., regularity of role hierarchies, whether roles used in cardinality constraints are simple, whether the used datatypes are OWL 2 datatypes, or whether a given lexical form corresponds to a data value in the value space of the specified datatype. Such issues are usually detected by the reasoners during parsing and, hence, are usually reported as parsing errors.

**Table 6:** Results for the user submitted ontologies (times are reported in seconds)

Reasoner	CO	DINTO	DPO	GO+
Consistency				
Chainsaw	1.80	4.86	0.76	6.05
FaCT++	2.32	*Error*	0.81	1254.24
HermiT	*Timeout*	9.68	2.98	202.42
HermiT-OA4	*Timeout*	12.66	3.64	71.52
Jfact	*Timeout*	*Timeout*	3.08	*Timeout*
Konclude	0.42	3.60	0.18	6.38
MORe	*Timeout*	*Error*	44.74	1103.87
Pellet-OA4	*Timeout*	35.53	2.55	*Error*
Racer	7.72	*Timeout*	0.93	79.88 (*wrong*)
TrOWL	4.10	10.94	2.38	38.02
Classification				
Chainsaw	*Timeout*	*Error*	5.82	*Timeout*
FaCT++	6652.28	*Error*	8.32	*Timeout*
HermiT	*Timeout*	*Error*	84.29	6227.97
HermiT-OA4	*Timeout*	*Error*	93.51	2351.83
Jfact	*Timeout*	*Timeout*	49.30 (*wrong*)	*Timeout*
Konclude	201.68	6.84	0.46	69.75
MORe	*Timeout*	*Error*	43.73	1023.11
Pellet-OA4	*Timeout*	*Error*	9.45	*Error*
Racer	*Timeout*	*Timeout*	*Timeout*	78.92 (*wrong*)
TrOWL	3.87 (*wrong*)	13.06	2.73 (*wrong*)	42.05 (*wrong*)

Italics indicate a failed attempt, either timeout, thrown error, or wrong (results)

**Table 7:** Results for the user submitted ontologies

Reasoner	VFB-EPNT	VFB-KB	VFB-NCT
Consistency			
Chainsaw	12.82	*Timeout*	12.71
FaCT++	10.20	*Timeout*	10.27
HermiT	11.11	*Timeout*	12.00
HermiT-OA4	10.43	*Error*	9.83
Jfact	121.92	*Timeout*	120.33
Konclude	3.73	3.81	3.93
MORe	*Error*	*Error*	*Error*
Pellet-OA4	9.06	*Error*	8.86
Racer	134.28 (*wrong*)	741.89 (*wrong*)	129.21 (*wrong*)
TrOWL	10.49	*Error*	9.10
Classification			
Chainsaw	*Error*	*Error*	*Error*
FaCT++	10.24	*Timeout*	9.93
HermiT	9.59	*Timeout*	9.19
HermiT-OA4	9.93	9.10 (*wrong*)	9.95
Jfact	121.71	9.22 (*wrong*)	121.38
Konclude	3.87	24.45	3.40
MORe	*Error*	*Error*	*Error*
Pellet-OA4	*Error*	*Error*	*Error*
Racer	132.82 (*wrong*)	768.53 (*wrong*)	132.41 (*wrong*)
TrOWL	8.93	*Error*	9.46
Realisation			
Chainsaw	*Error*	*Error*	*Error*
FaCT++	10.19	*Timeout*	10.04
HermiT	9.37	*Timeout*	10.07
HermiT-OA4	9.40	*Error*	9.77
Jfact	121.42	*Timeout*	124.17
Konclude	3.92	4.24	3.93
PAGOdA	*Error*	*Timeout*	*Error*
Pellet-OA4	*Error*	8.76	*Error*
Racer	160.64 (*wrong*)	812.88 (*wrong*)	134.55 (*wrong*)
TrOWL	8.89	*Error*	8.78

Italics indicate a failed attempt, either timeout, thrown error, or wrong (results)

Taking into account all user-submitted ontologies, i.e., submissions from 2014 and 2015 (see Table 8), every submission was processed by at least one reasoner. There were two ontologies that every DL reasoner classified successfully within the timeout: HP and DPC-OLY. And two ontologies that only one reasoner (TrOWL) terminated classification on: GALEN-FU and G-CDS; the remaining reasoners timed-out (there were five timeouts on GALEN-FU, and eight on G-CDS) or threw an error (four reasoners erred on GALEN-FU, and one on G-CDS). There were seven ontologies that were processed by all reasoners except Chainsaw. For consistency checking, there were nine ontologies successfully processed by every reasoner. The MSC-D ontology incurred the most timeouts; five reasoners did not complete the task. There were additional ontologies that were challenging to check consistency, such as all three versions of FHKB where at least one reasoner timed-out, both variants of GALEN, and all three versions of CB had two to three timeouts each. The realisation task had two ontologies (HP and DPC-OLY) as the only ones successfully processed by every reasoner, the exact same ones as during classification, and seven other ontologies that were processed by all but one reasoner (typically Chainsaw, which would throw some reasoner internal error). Several ontologies proved challenging for reasoners to complete realisation: both GALEN variants, MSC-D and MSC, on each of which five reasoners timed-out, and G-CDS where eight reasoners timed out. Konclude completed realisation on the most ontologies: 23. Similar to the other tasks, HermiT-OA4 processed more ontologies than HermiT, in particular exhibiting three timeouts less during realisation as well as consistency checking.

**Table 8:** Overall results for the user submitted ontologies with numbers in brackets showing %; sorting is by task, supported profile (EL reasoners are shown last) and % of solved problems; only ontologies with an ABox are used in the realisation track

Reasoner	Completed	Error	Timeout	All
Consistency				
Konclude	33 (91.67)	1 (2.78)	2 (5.56)	36
HermiT-OA4	29 (80.56)	5 (13.89)	2 (5.56)	36
HermiT	27 (75.00)	4 (11.11)	5 (13.89)	36
TrOWL	27 (75.00)	9 (25.00)	0 (0.00)	36
Chainsaw	25 (69.44)	8 (22.22)	3 (8.33)	36
Racer	24 (66.67)	0 (0.00)	12 (33.33)	36
Pellet-OA4	23 (63.89)	8 (22.22)	5 (13.89)	36
Jfact	22 (61.11)	9 (25.00)	5 (13.89)	36
FaCT++	21 (58.33)	12 (33.33)	3 (8.33)	36
MORe	19 (52.78)	9 (25.00)	8 (22.22)	36
ELepHant	1 (100.00)	0 (0.00)	0 (0.00)	1
ELK	1 (100.00)	0 (0.00)	0 (0.00)	1
jcel	1 (100.00)	0 (0.00)	0 (0.00)	1
Classification				
Konclude	29 (80.56)	2 (5.56)	5 (13.89)	36
TrOWL	27 (75.00)	9 (25.00)	0 (0.00)	36
HermiT-OA4	24 (66.67)	6 (16.67)	6 (16.67)	36
HermiT	23 (63.89)	6 (16.67)	7 (19.44)	36
Jfact	22 (61.11)	9 (25.00)	5 (13.89)	36
FaCT++	19 (52.78)	13 (36.11)	4 (11.11)	36
Racer	19 (52.78)	0 ( 0.00)	17 (47.22)	36
MORe	17 (47.22)	10 (27.78)	9 (25.00)	36
Pellet-OA4	14 (38.89)	9 (25.00)	13 (36.11)	36
Chainsaw	7 (19.44)	21 (58.33)	8 (22.22)	36
ELepHant	1 (100.00)	0 (0.00)	0 (0.00)	1
ELK	1 (100.00)	0 (0.00)	0 (0.00)	1
jcel	0 (0.00)	1 (100.00)	0 (0.00)	1
Realisation				
Konclude	23 (79.31)	3 (10.34)	3 (10.34)	29
TrOWL	21 (72.41)	8 (27.59)	0 (0.00)	29
Jfact	19 (65.52)	9 (31.03)	1 (3.45)	29
FaCT++	17 (58.62)	10 (34.48)	2 (6.90)	29
HermiT-OA4	17 (58.62)	7 (24.14)	5 (17.24)	29
HermiT	16 (55.17)	5 (17.24)	8 (27.59)	29
Racer	14 (48.28)	0 ( 0.00)	15 (51.72)	29
Pellet-OA4	13 (44.83)	9 (31.03)	7 (24.14)	29
Chainsaw	9 (31.03)	17 (58.62)	3 (10.34)	29

In terms of errors for the 2014 user-submitted corpus, FaCT++ and Chainsaw were unable to process GALEN-FU and GALEN-H due to unsupported datatypes, and in addition to this we identified the same types of errors as in the 2015 corpus, namely ontology parsing issues and reasoner internal errors.

## Conclusion

The ORE 2015 Reasoner Competition continued the success of its predecessors. Participants, workshop attendees, and interested bystanders all had fun, and the ORE 2015 corpus, whether used with the ORE framework or in a custom test harness, is a significant and distinct corpus for reasoner experimentation. Developers can easily rerun this year’s competition with new or updated reasoners to get a sense of their relative progress, and we believe that solving all the problems in that corpus in similar or somewhat relaxed time constraints is a reliable indicator of a very high quality implementation.

The top slots in all tracks have been dominated by Konclude (and to a lesser extent by ELK) for 2 years now. Konclude is a highly optimised, very efficient reasoner whose developers continuously test it against a vast set of available ontologies. Even so, there is interesting jockeying around second and third place for all tracks, and we were impressed with how well older reasoners, which have not been updated recently (notably Pellet-OA4 and Racer), fared. Both across user-submitted ontologies (6 h timeout) and the live competition (3 min timeout), (almost) every ontology was processed by at least one reasoner. This is a considerable result for the community overall.

Given this stasis in results, we have decided to move to a 2-year cycle for competitions. This allows more time for reasoners and the corpus to develop, as well as giving us more resources to develop additional tracks. It is possible that Konclude will remain the champion, which we regard as challenge for the competition. We are experimenting with different biases in our problem selection (e.g., favouring difficult problems) to increase the competitiveness of the corpus. Adding additional tracks will also potentially ameliorate this problem.

The robustness experiments in [10] used a much longer timeout (up to 2 h per test), though the analysis clustered results by subdivisions of the timeout period. That suggests that a slightly longer timeout might significantly increase the total number of solved problems across reasoners. Increasing the timeout needs to be weighed against the increased overall run time of the competition (which is bounded by the slowest reasoner). We prefer the bulk of the competition to be executed during a single day of the DL workshop to facilitate engagement. This imposes fairly tight limits on the timeout and number of problems.^24^ Moreover, since almost all ontologies were processed by at least one reasoner in the ORE 2015 competition, we believe that our current setting is reasonably well balanced. Our offline competition remains a suitable reasoner benchmark (with a longer timeout) using difficult ontologies from users in need of our services.

Ideally, the ORE toolkit and corpora will serve as a nucleus for an infrastructure for common experimentation. To that end, results and analysis scripts are made available online.^25^ The test harness seems perfectly well suited for black box head-to-head comparisons, and we recommend experimenters consider it before writing a home grown one. This will improve the reliability of the test harness as well as reproducibility of experiments. Even for cases where more elaborate internal measurements are required, the ORE harness can serve as the command and control mechanism. For example, separating actual calculus activity from other behavior (parsing, serializing, etc.) requires a deep delve into the reasoner internals. However, given a set of reasoners that could separate out those timings, it would be a simple extension to the harness to accommodate them.

While the test harness works well for “head-to-head, fire-and-forget” experiments, the analysis scripts are more tuned for competition and not experimentation. For example, an experiment can have two reasoners that solve all problems within the timeout, but one is twice as fast as the other. Most algorithm and implementation comparisons will want to delve into that fact. There is no consensus of how to do such analysis at the moment, but it would be straightforward to add additional analysis scripts (for example, Fig. 3 was generated from standard ORE data by custom scripts).

The ORE *workshop* solicits “challenge” ontologies from ontology developers partly in the hopes of directing reasoner developer attention to real user performance needs. In 2005, we have, for the first time, incorporated an (offline) challenge involving user submitted ontologies. While the results do not count towards the overall rankings of the reasoners, we hope that they provide guidance for ontology users to select appropriate reasoners for their problems and, perhaps, serve as an incentive for reasoner developers to develop better optimisations.

The most important next expansion of tracks is to conjunctive query answering (CQA). Setting up a meaningful CQA competition is significantly more difficult, because we do not only have to consider ontologies, but also queries and data. Gathering suitable (meaningful) queries is probably the most difficult hurdle to overcome. However, we made significant progress toward a reasonable design this year and hope to incorporate it in the next competition.

Another area of interest is application-style benchmarks, which would situate the reasoning task in the context of a pattern of use that is characteristic of a real or realistic application. This might include modification of the ontology or data during the competition run.

Our current dispute resolution mechanism is unsatisfactory. Recent work [19] has revealed examples *in the 2015 corpus* where the correct reasoner would be unfairly penalized for being in the minority. Furthermore, incomplete reasoners (or unsound ones, should any come forward) remain a problem. Our solution, in development [19], combines a more sophisticated voting procedure with select manual verification. We hope to incorporate the manual verification step as a form of “bystander” participation (in addition to the results-prediction competition).

There are still challenges in constructing a meaningful corpus that allows for generalisation or proper reasoner comparison, in particular, since reasoners do not typically implement exactly the same fragments of OWL (notably, datatype support varies widely, and most EL reasoners implement slightly different subsets of OWL EL). In some respects, designing a corpus for a competition is easier in that, in the end, the results of a competition are just that...the outcomes of a contest.

## Footnotes

1 The websites for DL2015 and ORE2015 are archived at http://dl.kr.org/dl2015/ and https://www.w3.org/community/owled/ore-2015-workshop/ respectively.

2
http://owl.cs.manchester.ac.uk/publications/supporting-material/ore-2015-report/.

3 See the CASC website for details on past competitions: http://www.tptp.org. Also of interest, though not directly inspirational for ORE, is the SAT competition http://www.satcompetition.org.

4
http://www.cs.ox.ac.uk/isg/ontologies/.

5 Which may be exceeded considering that all reasoners in the competition run in parallel.

6 Duplicates are those that are *byte identical* after being “DLified” and serialised into Functional Syntax.

7 We have included the user submitted ontologies of 2014 because we neglected to evaluate them separately at the time of the 2014 competition.

8 Submitted by Dr. David Osumi-Sutherland, GO Editorial Office, European Bioinformatics Institute, European Molecular Biology Laboratory, Wellcome Trust Genome Campus, Hinxton, Cambridge, UK.

9 Available at https://github.com/obophenotype/cell-ontology.

10 Submitted by María Herrero, Computer Science Department, Univesidad Carlos III de Madrid. Leganés, Spain.

11 All versions are available at https://code.google.com/archive/p/dinto/.

12 Available at https://github.com/FlyBase/flybase-controlled-vocabulary.

13 Available at http://purl.bioontology.org/ontology/GO-PLUS.

14 All ontologies are available at https://github.com/VirtualFlyBrain.

15 Available at https://github.com/ghxiao/city-bench.

16 Available at http://www.e-lico.eu/DMOP.html.

17 Available at https://code.google.com/archive/p/care-engine/downloads.

18 Available at https://bioportal.bioontology.org/ontologies/GENE-CDS.

19 Available at https://github.com/TheOntologist/FHKB.

20
https://github.com/andreas-steigmiller/ore-competition-framework/. A detailed description of the framework and how to run it is available there.

21 Due to the majority voting, a tight time limit could potentially benefit reasoners that guess or approximate results if sound and complete reasoners cannot determine the correct ones within the time limit. However, test runs of the competition have revealed that a moderately increased time limit (e.g., 300 s instead of 150 s) does not seem to significantly influence the overall results, i.e., most reasoners cannot solve many additional problems if the time limit is only moderately increased. These test runs further showed that 30 s are (usually) enough to parse the ontologies.

22
http://owl.cs.manchester.ac.uk/tools/list-of-reasoners/.

23 Upon closer inspection, Racer actually throws a *stack overflow* error in these cases, but the error is not piped out to the framework’s error handler. As a consequence, the wrong result is only pinned down upon correctness checking rather than flagged as an error straight away.

24 Unfortunately this year, due to technical issues, we had to run the competition over night.

25
http://owl.cs.manchester.ac.uk/publications/supporting-material/ore-2015-report/.
